# Adherence to the Korean National Code Against Cancer and mortality: a prospective cohort study from the Health Examinees-Gem study

**DOI:** 10.4178/epih.e2025026

**Published:** 2025-05-09

**Authors:** Jeeyoo Lee, Aesun Shin, Woo-Kyoung Shin, Ji-Yeob Choi, Daehee Kang

**Affiliations:** 1 Department of Preventive Medicine, Seoul National University College of Medicine, Seoul, Korea; 2 Cancer Research Institute, Seoul National University, Seoul, Korea; 3 lnterdisciplinary Program in Cancer Biology Major, Seoul National University College of Medicine, Seoul, Korea; 4 lntegrated Major in Innovative Medical Science, Seoul National University Graduate School, Seoul, Korea; 5 Medical Research Center, Genomic Medicine Institute, Seoul National University College of Medicine, Seoul, Korea; 6 Division of Food and Pharmaceutical Technology, Mokwon University, Daejeon, Korea; 7 Department of Biomedical Science, Seoul National University Graduate School, Seoul, Korea; 8 Institute of Health Policy and Management, Seoul National University Medical Research Center, Seoul, Korea

**Keywords:** Cancer, Korea, Guideline, Mortality

## Abstract

**OBJECTIVES:**

The Korean National Code Against Cancer was released in 2006. These guidelines aimed to promote a healthy lifestyle to prevent cancer risk through 10 recommendations. The objective of this study was to investigate the associations between adherence to the Korean National Code Against Cancer and the risk of all-cause, cancer, and cardiovascular disease (CVD) mortality among Koreans.

**METHODS:**

This prospective cohort study included 109,160 Korean adults aged 40 years to 69 years, recruited from 2004 to 2013 in the population-based Health Examinees-Gem Study. The adherence total score was calculated based on 6 items from the Korean National Code Against Cancer: smoking, consuming vegetables and fruits, limiting salty foods, restricting alcohol intake, engaging in physical activity, and maintaining a healthy weight. Hazard ratios (HRs) and 95% confidence intervals (CIs) for the associations of adherence scores with mortality risk were estimated using a Cox proportional hazards regression model.

**RESULTS:**

During a mean follow-up period of 12.0 years, 3,799 deaths were recorded. According to the multivariable-adjusted model, males in the highest tertile of adherence scores had a lower risk of all-cause, cancer, and CVD mortality compared to those in the lowest tertile (all-cause: HR, 0.67; 95% CI, 0.60 to 0.74; cancer: HR, 0.63; 95% CI, 0.54 to 0.74; CVD: HR, 0.56, 95% CI, 0.43 to 0.73). A similar association was observed among females for all-cause and CVD mortality (all-cause: HR, 0.85; 95% CI, 0.76 to 0.96; CVD: HR, 0.70; 95% CI, 0.51 to 0.97).

**CONCLUSIONS:**

Adherence to the Korean National Code Against Cancer was associated with a reduced risk of all-cause, cancer, and CVD mortality.

## GRAPHICAL ABSTRACT

[Fig f1-epih-47-e2025026]

## INTRODUCTION

Cancer is a leading cause of death worldwide [1], with lifestyle factors such as smoking, excessive alcohol consumption, physical inactivity, obesity, and unhealthy dietary habits significantly contributing to cancer risk. Previous literature suggests that approximately 30-50% of cancer cases are preventable [2,3] through lifestyle modifications, prompting various international organizations to develop cancer prevention guidelines. Notable examples include Research (WCRF/AICR) recommendations [4], the American Cancer Society guidelines [5], and the European Code against Cancer [6]. Similarly, Japan has introduced 6 key cancer prevention rules [7]. These recommendations commonly emphasize a healthy diet, regular physical activity, and the avoidance of tobacco and alcohol consumption. In Korea, the Ministry of Health and Welfare recognized the importance of cancer prevention and introduced the first edition of the Korean National Code Against Cancer in 2006, emphasizing the adoption of healthy habits in daily life [8]. A major revision released in 2016 presented 10 key recommendations: smoking cessation, increased consumption of vegetables and fruits, reduced salt intake, limited alcohol consumption, regular physical activity, maintenance of a healthy weight, vaccination, sexual health, occupational safety, and participation in cancer screening [9]. This code was specifically tailored to the Korean population, considering cultural and dietary lifestyle patterns.

Non-communicable diseases, including cancer and cardiovascular disease (CVD), account for 74% of global deaths [10]. In Korea, cancer remains the leading cause of death, responsible for 24.2% of all deaths in 2023 [11]. Given the high burden of cancer in Korea, lifestyle-based prevention strategies are essential for reducing mortality risk.

Previous studies have shown that adherence to the 2018 WCRF/AICR cancer prevention recommendations was associated with lower mortality in older adults in the United States [12] and Switzerland [13]. Epidemiological studies conducted in various countries have consistently reported an association between healthy lifestyle habits (e.g., limiting alcohol consumption, smoking cessation, regular physical activity, maintaining a healthy weight, and a balanced diet) and reduced mortality risk [14-23]. Cancer prevention guidelines are designed to reduce cancer risk, and previous studies have confirmed their effectiveness [24]. Additionally, studies have reported that adherence to healthy lifestyle habits outlined in these guidelines also modifies the risk of other major causes of death, such as CVD [12]. Previous studies on lifestyle factors and mortality have analyzed various datasets [15,16,18,22]. One Korean study investigated associations between individual lifestyle factors and both cancer incidence and mortality using data from the National Health Insurance Service-Health Screening cohort; however, dietary factors were not considered [18]. In this study, we investigated the associations between adherence scores based on the Korean National Code Against Cancer and the risks of all-cause, cancer, and CVD mortality within a large Korean cohort.

## MATERIALS AND METHODS

### Study population

The Health Examinees Study (HEXA) is a comprehensive prospective cohort study conducted on a population scale, aiming to identify the genetic and environmental determinants of prevalent chronic diseases. This study is part of the Korea Genomic Epidemiology Study (KoGES), which is managed by the Korea Disease Control and Prevention Agency. From 2004 to 2013, 173,195 individuals participated at 38 hospitals and regional health examination centers in eight regions across the country. This study strictly adhered to a standardized research protocol, which has been detailed extensively in previous literature [25].

The Health Examinees-Gem Study (HEXA-G) included 139,263 participants aged 40-69 years and applied supplementary eligibility criteria for institutions involved in the HEXA [26]. Individuals who did not agree to linkage with death certificates (n=23,212), lacked dietary information from the food frequency questionnaire (n=1,379), had implausible levels of energy intake (<500 or >4,000 kcal/day; n=797), or had missing data for the main variables derived from the Korean cancer prevention guidelines (body mass index [BMI], waist circumference, physical activity, smoking status; n=4,715) were excluded. The final analysis included 109,160 participants.

### The Korean National Code Against Cancer score construction

Data on participants’ demographic characteristics, lifestyle habits, and medical histories were collected at baseline using a structured questionnaire. Dietary intake was assessed using a validated 106-item semi-quantitative food frequency questionnaire [27]. Food consumption and daily energy intake were calculated using the food composition table developed by the Korean Health Industry Development Institute [28].

We scored 6 out of the 10 published recommendations: (1) not smoking, (2) consuming plenty of vegetables and fruits, (3) consuming foods low in salt, (4) avoiding drinking alcohol, (5) engaging in regular physical activity, and (6) maintaining a healthy weight. Four recommendations (related to vaccination, sexual health, occupational safety and health, and cancer screening) were excluded from analysis, as they could not be measured. Our scoring method was based on the approach used by Shams-White et al. [29] in a previous study that utilized similar items from the 2018 WCRF/AICR cancer prevention recommendations and the Korean National Code Against Cancer (fruit and vegetable intake, alcohol restriction, and physical activity) [24]. For the smoking category, scores were assigned as follows: never smokers received 1.0 point, former smokers received 0.5 points, and current smokers received 0.0 points. For the salty food category, sodium intake was used as the criterion. The scoring for sodium intake in our study was based on the Dietary Reference Intakes for Koreans 2020 [30], which referenced the U.S. Dietary Reference Intake report and its dose-response meta-analysis [31]. This analysis categorized sodium intake into 3 levels (<2,300, 2,300-4,100, and >4,100 mg/day) and found that intake within the range of 2,300-4,100 mg/day was associated with the lowest risk of CVD and hypertension. Additionally, some reports suggest sodium intake below 700 mg/day may increase insulin resistance. Based on this evidence, we applied the following scoring system: a score of 1.0 was assigned for an intake between 2,300-4,100 mg/day, 0.5 points for an intake of 700-2,300 mg/day or 4,100-5,000 mg/day, and 0.0 points for an intake below 700 mg/day or above 5,000 mg/day. BMI and abdominal obesity criteria were based on standards established by the Korean Society for the Study of Obesity. A score of 1.0 point was given for full compliance with the recommendation, 0.5 points for partial compliance, and 0.0 points for non-compliance. Higher scores indicated greater adherence to the Korean National Code Against Cancer. Each component of the scoring system is detailed in Supplementary Material 1.

### Ascertainment of causes of death

Deaths occurring between baseline and December 31, 2021, were verified using the death certificate database from the National Statistical Office of Korea, along with unique identifiers such as resident registration numbers. Causes of death were categorized according to the 10th revision of the International Classification of Diseases (ICD-10). Total mortality included deaths from all causes, cancer mortality included deaths classified under ICD codes C00-C97 and D00-D48, and CVD mortality included deaths classified under ICD codes I00-I99. Cancer-specific mortality was defined using ICD codes C33-C34 (lung cancer), C16 (stomach cancer), C18-C20 (colorectal cancer), C50 (breast cancer), and C61 (prostate cancer).

### Statistical analysis

Based on previous literature and the distribution of adherence scores [12,24], scores were categorized into tertiles. Participant characteristics at baseline according to adherence score category and sex were expressed as percentages for categorical variables and as means with standard deviations for continuous variables. General characteristics were compared based on adherence to the Korean National Code Against Cancer using the chi-square test for categorical variables and generalized linear models for continuous variables. Trends were assessed by treating the median value of each adherence score category as a continuous variable.

We used Cox proportional hazards regression models to estimate hazard ratios (HRs) and 95% confidence intervals (CIs) for associations between tertiles of adherence scores to the Korean National Code Against Cancer and mortality risk. Age was used as the time scale; participants entered the study at the age when they completed the baseline questionnaire and exited at the age of death or the last follow-up date (December 31, 2021), whichever occurred first. The proportional hazards assumption was assessed using the Schoenfeld residuals method, and no violations were detected (p>0.05 for all). Based on previous literature [12,24], we adjusted for potential confounder, including education level (less than high school, high school, college or more, and missing), total energy intake (tertiles), and Charlson comorbidity index (continuous) [32]. Missing data for categorical covariates were treated as a separate dummy category in the multivariable Cox proportional hazards regression models. To minimize potential reverse causality from undiagnosed prevalent conditions at baseline, we performed a sensitivity analysis that excluded the initial 2 years of follow-up. All analyses were performed using SAS version 9.4 (SAS Institute Inc., Cary, NC, USA).

### Ethics statement

The Ethics Committee of KoGES at the Korea National Institute of Health (IRB No. 2014-08-02-3C-A) and the Institutional Review Board of Seoul National University Hospital in Seoul, Korea (IRB No. E-2309-038-1464), approved the study protocol, and signed consent forms were obtained from all participants.

## RESULTS

The median adherence score was 3.00 (interquartile range, 2.50-3.75) for male and 4.00 (interquartile range, 3.50-4.50) for female. During a mean follow-up period of 12.0 years, a total of 3,799 deaths were identified, including 2,249 male and 1,550 female. Table 1 summarizes the demographic characteristics of the study participants categorized by adherence to the Korean National Code Against Cancer. Individuals with higher adherence scores to the Korean National Code Against Cancer generally exhibited higher levels of physical activity, sodium intake, fruit and vegetable consumption, and total energy intake. Additionally, those with higher scores had a greater proportion of never-smokers, lower BMI, and reduced alcohol consumption compared to those with lower adherence scores.

Table 2 presents the risk of all-cause, cancer, and CVD mortality according to adherence to the Korean National Code Against Cancer. In adjusted models, male in the highest adherence score category had a significantly reduced risk of all-cause mortality (HR, 0.67; 95% CI, 0.60 to 0.74), cancer mortality (HR, 0.63; 95% CI, 0.54 to 0.74), and CVD mortality (HR, 0.56; 95% CI, 0.43 to 0.73). Female who adhered more closely to the Korean National Code Against Cancer showed a 15% decrease in risk of all-cause mortality (HR: 0.85, 95% CI: 0.76 to 0.96) and a 30% decrease in risk of CVD mortality (HR, 0.70; 95% CI, 0.51 to 0.97). Supplementary Material 2 provides additional data on the association between cancer-specific mortality. Male with the highest adherence had a 50% reduced risk of lung cancer mortality (HR, 0.50; 95% CI, 0.37 to 0.69).

Table 3 and Supplementary Materials 3-7 detail the associations between adherence to individual components of the Korean National Code Against Cancer adherence score and risks of all-cause mortality, cancer mortality, and cancer-specific mortality (lung, stomach, colorectal, prostate, and breast cancers). Among never-smokers, the risk of all-cause mortality was reduced by 51% in male and 54% in female compared with current smokers (male: HR, 0.49; 95% CI, 0.44 to 0.55; female: HR, 0.46; 95% CI, 0.35 to 0.59). Additionally, never-smokers had a 58% lower risk of cancer mortality in male and a 48% lower risk in female (male: HR, 0.42; 95% CI, 0.35 to 0.49; female: HR, 0.52; 95% CI, 0.36 to 0.76). The risk of lung cancer mortality was reduced by 87% in male and 78% in female who never smoked compared to current smokers (male: HR, 0.13; 95% CI, 0.09 to 0.21; female: HR, 0.22; 95% CI, 0.11 to 0.44). Male engaging in more than 150 minutes of exercise per week had a 23% lower risk of all-cause mortality (HR, 0.77; 95% CI, 0.70 to 0.84), and female had an 11% lower risk (HR, 0.89; 95% CI, 0.80 to 0.99) compared to those exercising less than 75 minutes per week. Similar risk reductions were observed for cancer and lung cancer mortality. Interestingly, male with normal BMI had a 21% higher risk of all-cause mortality compared to those categorized as underweight or obese (HR, 1.21; 95% CI, 1.10 to 1.33), a pattern was also observed for cancer and lung cancer mortality.

A sensitivity analysis excluding the first 2 years of follow-up included 37,224 male and 71,638 female (Supplementary Material 8). The finding were consistent with the primary analysis.

## DISCUSSION

This prospective cohort study showed an inverse association between adherence to the Korean National Code Against Cancer and the risks of all-cause, cancer, and CVD mortality among Korean male. Similar patterns were observed in female for all-cause and CVD mortality. The associations varied by sex and cause of death. Our findings emphasize the importance of adherence to the Korean National Code Against Cancer not only in cancer prevention but also in reducing overall mortality.

To our knowledge, no previous study has examined associations between adherence to the Korean National Code Against Cancer and the risks of all-cause, cancer, and CVD mortality. A prior study adopting the Korean National Code Against Cancer, using a scoring method identical to ours, examined cancer incidence rather than mortality [24]. Previous studies [15,16,22], including large-scale epidemiological studies in Korea such as the Korea National Health and Nutrition Examination Survey and the Korean Longitudinal Study of Aging, investigated the associations between lifestyle factor scores and mortality. Most of these studies found that higher lifestyle scores, indicating healthier behaviors, were associated with a lower mortality risk. Similar results have been reported from studies conducted in the United States [20], Europe [14], China [17,21], and Japan [19]. While direct comparisons with other studies are difficult due to differences in measured lifestyle factors, most research suggests that adherence to healthy lifestyle habits significantly reduces mortality risk.

Sodium, consumed primarily as salt, is a physiologically essential nutrient [33]. Excessive sodium intake is associated with increased risks of hypertension and CVD [34]. Many dietary guidelines advocate reducing sodium intake; for example, the World Health Organization (WHO) recommends that adults limit daily sodium intake to less than 2,000 mg [35]. However, insufficient sodium intake (below 700 mg/day) may cause clinical problems such as increased insulin resistance [36] and elevated total cholesterol levels [37]. Studies investigating the association between sodium intake and mortality have produced conflicting results [38]. Contrary to the widespread belief that lower sodium intake is always healthier, recent studies report increased mortality associated with both excessively low-sodium and high-sodium diets [39]. A recent analysis involving 140,000 Koreans found no clear association between sodium intake and mortality, suggesting that the impact of sodium intake on disease and mortality risk may vary by race, region, and dietary habits [40]. Currently, there are no precise numerical guidelines to determine optimal sodium intake associated with the lowest mortality risk [41]. Therefore, we established reference points for sodium scores based on findings from a meta-analysis conducted by the Sodium Committee of the U.S. Dietary Reference Intakes [31]. We recognize that these guidelines may need revision as additional evidence becomes available.

Smoking is a major risk factor for chronic diseases, including cancer, and a leading cause of preventable premature death [42]. Our study demonstrated that male and female who never smoked had more than a 50% lower mortality risk compared to current smokers. Specifically, the risk of lung cancer mortality was reduced by over 80%. Studies conducted in several other countries have similarly reported increased mortality associated with smoking [43,44]. Although the smoking rate among Korean male decreased from 48.3% in 2010 to 32.4% in 2023, smoking among young Korean female is increasing [45]. Based on our findings and previous research, efforts to promote smoking cessation are essential for mortality reduction.

Individual factor analysis based on BMI revealed that normal-weight male had a 21% higher risk of all-cause mortality compared to underweight or obese male. Similar patterns were observed for cancer and lung cancer mortality. A previous Korean study [46] reported that individuals with a BMI of 21.5-27.9 kg/m^2^ had the lowest overall mortality risk, consistent with results from a large-scale Asian population study [47]. These findings suggest that the BMI criteria recommended by the WHO Asia-Pacific Region and the Korean Society for the Study of Obesity might not be fully appropriate for Koreans. Japan and Korea both define obesity using a BMI threshold of 25 kg/m^2^, while China uses 28 kg/m^2^ [48]. Importantly, while BMI is widely used as a simple indicator of obesity in epidemiological studies, it alone is insufficient for a clinical diagnosis of obesity. Further studies utilizing various obesity measurement tools are necessary to clarify the association between obesity and mortality and to identify more appropriate BMI standards for the Korean population.

The traditional Korean diet typically consists of *bab* (cooked rice), *kuk* (broth-based dishes), and various *banchans* (side dishes), always including kimchi [49]. This diet is characterized by high vegetable intake, facilitated by Korea’s advanced agricultural practices and distinct seasonal variations. Vegetables, consumed primarily as *banchans*, include various seasoned dishes (*namul*) and multiple kimchi varieties. A notable feature of Korean cuisine is the frequent use of seasonings such as green onions, red peppers, garlic, and ginger. High consumption of vegetables and fruits is widely reported to have numerous health benefits. Our study found that female consuming over 400 g/day of fruits and vegetables had a 16% lower mortality risk compared to those consuming less than 200 g/day. However, the proportion of Koreans consuming more than 500 g of fruits and vegetables per day has recently declined [50], and our analysis showed that only 16.2% of males and 14.7% of females consumed more than 400 g daily. Reversing this downward trend and encouraging dietary habits that increase vegetable and fruit intake is critically important.

Our study has several limitations to consider. First, due to data limitations, we could not incorporate all components of the Korean National Code Against Cancer. Future studies should aim to include additional guideline components. Second, our analysis relied on baseline measurements, assuming participants maintained the same lifestyle throughout the study period. Although no violations of proportional hazard assumptions were observed, lifestyle factors may have changed over time. Predictive modeling could potentially enhance the assessment of lifestyle factors by allowing time-dependent risk estimations, but this approach was beyond our current scope and should be addressed in future research. Third, although we adjusted for numerous confounding factors, residual and unmeasured confounders may still be present. Fourth, although cancer-specific mortality was analyzed for lung, stomach, colorectal, prostate, and breast cancers, the limited number of cases reduced statistical power, necessitating cautious interpretation. Despite these limitations, our study offers a comprehensive evaluation of the associations between adherence to the Korean National Code Against Cancer and mortality risk using a large-scale cohort study. Death confirmation within this cohort was validated by the National Statistical Office of Korea, indicating a high level of accuracy in death diagnosis.

In conclusion, higher adherence to the Korean National Code Against Cancer was confirmed to be beneficial for reducing mortality among Koreans. Promoting awareness of and adherence to the Korean National Code Against Cancer is crucial. Although primarily focused on cancer prevention, these guidelines could also serve as an effective tool for reducing overall mortality in Korea.

## Figures and Tables

**Figure f1-epih-47-e2025026:**
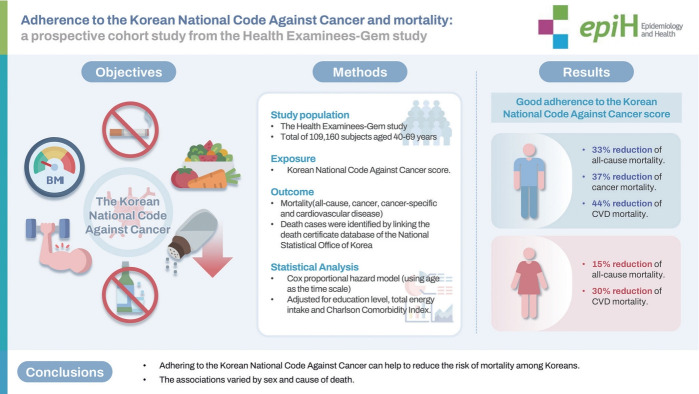


**Table 1.** Baseline characteristics of the participants according to Korean National Code Against Cancer adherence score categories and sex

Characteristics	Male (n=37,414)			p-value^1^	Female (n=71,746)			p-value^1^
	Tertile 1	Tertile 2	Tertile 3		Tertile 1	Tertile 2	Tertile 3	
Score range	0.00-2.50	2.75-3.50	3.75-6.00		0.50-3.50	3.75-4.25	4.50-6.00	
No. of participants	12,121 (32.4)	13,965 (37.3)	11,328 (30.3)		28,764 (40.1)	18,986 (26.5)	23,996 (33.5)	
Age (yr)	52.3±8.3	53.7±8.4	55.2±8.3	<0.001	52.4±8.0	52.4±7.7	52.4±7.5	0.674
Body mass index (kg/m^2^)	25.3±3.0	24.3±2.7	23.5±2.2	<0.001	24.5±3.4	23.5±2.4	22.6±2.4	<0.001
Education				<0.001				<0.001
≤Middle school	2,775 (22.9)	2,805 (20.1)	2,076 (18.3)		11,729 (40.8)	6,765 (35.6)	7,109 (29.6)	
High school	5,142 (42.4)	5,751 (41.2)	4,353 (38.4)		11,571 (40.2)	8,292 (43.7)	11,201 (46.7)	
≥College	4,087 (33.7)	5,299 (37.9)	4,799 (42.4)		5,225 (18.2)	3,788 (20.0)	5,522 (23.0)	
Missing	117 (1.0)	110 (0.8)	100 (0.9)		239 (0.8)	141 (0.7)	164 (0.7)	
Income (10^4^ Korean won)				0.327				<0.001
<200	2,973 (24.5)	3,372 (24.2)	2,706 (23.9)		9,929 (34.5)	5,468 (28.8)	6,041 (25.2)	
200-400	5,233 (43.2)	6,067 (43.4)	4,826 (42.6)		10,773 (37.5)	7,586 (40.0)	9,748 (40.6)	
≥400	3,266 (26.9)	3,797 (27.2)	3,145 (27.8)		6,002 (20.9)	4,586 (24.2)	6,429 (26.8)	
Missing	649 (5.4)	729 (5.2)	651 (5.8)		2,060 (7.2)	1,346 (7.1)	1,778 (7.4)	
Smoking status				<0.001				<0.001
Never	1,054 (8.7)	3,455 (24.7)	5,560 (49.1)		26,628 (92.6)	18,686 (98.4)	23,879 (99.5)	
Former	4,255 (35.1)	6,460 (46.3)	4,749 (41.9)		653 (2.3)	174 (0.9)	79 (0.3)	
Current	6,812 (56.2)	4,050 (29.0)	1,019 (9.0)		1,483 (5.2)	126 (0.7)	38 (0.2)	
Alcohol intake (g of ethanol/day)	24.1±43.2	13.9±26.0	7.1±17.0	<0.001	3.3±27.7	1.4±5.1	0.7±3.3	<0.001
Physical activity (min/wk)				<0.001				<0.001
<75	9,371 (77.3)	6,459 (46.3)	1,884 (16.6)		23,833 (82.9)	9,622 (50.7)	4,816 (20.1)	
75-<150	946 (7.8)	1,503 (10.8)	1,006 (8.9)		1,970 (6.9)	2,292 (12.1)	2,227 (9.3)	
≥150	1,804 (14.9)	6,003 (43.0)	8,438 (74.5)		2,961 (10.3)	7,072 (37.3)	16,953 (70.7)	
Energy intake (kcal/day)	1,773.7±512.7	1,846±488.6	1,912.8±478.0	<0.001	1,564.8±485.8	1,718.5±509.4	1,809.6±494.2	<0.001
Vegetable and fruits intake (g/day)	214.6±176.0	270.0±178.9	326.0±169.2	<0.001	191±157.1	272.9±181.6	334.1±169.3	<0.001
Sodium intake (g/day)	2,263.1±1,514.7	2,665.5±1,411.9	3,025.8±1,209.3	<0.001	1,878.1±1,281.3	2,512.1±1,379.7	2,910.1±1,115.8	<0.001

Variables are presented as number (%) or mean ± standard deviation.

1 Using chi-square test (categorical variables) and generalized linear model (continuous variables).

**Table 2.** Hazard ratios (HRs) and 95% confidence intervals (CIs) for all-cause, cancer, and cardiovascular disease (CVD) mortality according to Korean National Code Against Cancer adherence score categories

Adherence score (range)	Person year	No. of total subjects	All-cause mortality			Cancer mortality			CVD mortality		
			No. of deaths	Crude HR (95% CI)	Adjusted HR (95% CI)^1^	No. of deaths	Crude HR (95% CI)	Adjusted HR (95% CI)^1^	No. of deaths	Crude HR (95% CI)	Adjusted HR (95% CI)^1^
Male (n=37,414)											
Tertile 1 (0.00-2.50)	144,022.5	12,121	823	1.00 (reference)	1.00 (reference)	383	1.00 (reference)	1.00 (reference)	144	1.00 (reference)	1.00 (reference)
Tertile 2 (2.75-3.50)	166,685.0	13,965	799	0.74 (0.67, 0.81)	0.76 (0.69, 0.84)	368	0.73 (0.63, 0.84)	0.74 (0.64, 0.85)	127	0.66 (0.52, 0.84)	0.68 (0.54, 0.87)
Tertile 3 (3.75-6.00)	135,252.1	11,328	627	0.62 (0.56, 0.69)	0.67 (0.60, 0.74)	290	0.61 (0.53, 0.72)	0.63 (0.54, 0.74)	95	0.53 (0.41, 0.69)	0.56 (0.43, 0.73)
p trend				<0.001	<0.001		<0.001	<0.001		<0.001	<0.001
Continuous (per 1-unit increse in score)				0.82 (0.79, 0.86)	0.85 (0.81, 0.89)		0.82 (0.77, 0.87)	0.83 (0.78, 0.89)		0.78 (0.70, 0.86)	0.80 (0.72, 0.89)
Female (n=71,746)											
Tertile 1 (0.50-3.50)	346,824.9	28,764	700	1.00 (reference)	1.00 (reference)	374	1.00 (reference)	1.00 (reference)	114	1.00 (reference)	1.00 (reference)
Tertile 2 (3.75-4.25)	229,565.5	18,986	385	0.86 (0.76, 0.98)	0.87 (0.77, 0.99)	222	0.92 (0.78, 1.08)	0.91 (0.77, 1.07)	60	0.84 (0.62, 1.15)	0.89 (0.65, 1.22)
Tertile 3 (4.50-6.00)	289,706.3	23,996	465	0.84 (0.75, 0.94)	0.85 (0.76, 0.96)	276	0.92 (0.78, 1.07)	0.89 (0.76, 1.05)	55	0.62 (0.45, 0.86)	0.70 (0.51, 0.97)
p trend				0.002	0.008		0.247	0.150		0.004	0.035
Continuous (per 1-unit increse in score)				0.90 (0.85, 0.95)	0.90 (0.85, 0.96)		0.95 (0.88, 1.03)	0.94 (0.87, 1.02)		0.78 (0.68, 0.91)	0.83 (0.72, 0.96)

1 Adjusted for education level (less than high school, high school, college or above and missing), Charlson comorbidity index (continuous), and total energy intake (tertiles).

**Table 3.** Associations between adherence to individual components of the Korean National Code Against Cancer and all-cause mortality

Components of Korean National Code Against Cancer score	Male (n=37,414)				Female (n=71,746)			
	No. of deaths/total participants	Person year	Crude HR (95% CI)	Adjusted HR (95% CI)^1^	No. of deaths/total participants	Person year	Crude HR (95% CI)	Adjusted HR (95% CI)^1^
Smoking status								
0.00	848/11,881	140,682.0	1.00 (reference)	1.00 (reference)	56/1,647	19,434.1	1.00 (reference)	1.00 (reference)
0.50	907/15,464	183,111.9	0.57 (0.52, 0.63)	0.57 (0.52, 0.63)	31/906	10,794.9	0.85 (0.54, 1.33)	0.85 (0.55, 1.32)
1.00	494/10,069	122,165.7	0.47 (0.42, 0.53)	0.49 (0.44, 0.55)	1,463/69,193	835,867.7	0.45 (0.34, 0.59)	0.46 (0.35, 0.59)
Eat plenty of vegetables and fruits								
0.00	942/14,612	173,614.7	1.00 (reference)	1.00 (reference)	691/30,120	361,181.5	1.00 (reference)	1.00 (reference)
0.50	965/16,727	198,806.0	0.91 (0.84, 1.00)	0.93 (0.85, 1.02)	654/31,056	373,488.7	0.94 (0.85, 1.05)	0.94 (0.85, 1.06)
1.00	342/6,075	73,538.9	0.88 (0.78, 1.00)	0.92 (0.81, 1.05)	205/10,570	131,426.5	0.82 (0.70, 0.96)	0.84 (0.71, 0.99)
Eat food without salty								
0.00	225/3,421	41,618.3	1.00 (reference)	1.00 (reference)	150/6,274	78,129.5	1.00 (reference)	1.00 (reference)
0.50	1,099/17,923	212,675.3	0.97 (0.84, 1.12)	0.97 (0.84, 1.12)	849/38,247	458,538.5	1.01 (0.85, 1.20)	1.01 (0.85, 1.21)
1.00	925/16,070	191,666.0	0.94 (0.82, 1.09)	0.96 (0.83, 1.11)	551/27,225	329,428.8	0.93 (0.78, 1.12)	0.96 (0.80, 1.15)
Limit alcohol consumption								
0.00	373/6,543	77,728.2	1.00 (reference)	1.00 (reference)	36/2,294	27,308.6	1.00 (reference)	1.00 (reference)
0.50	1,073/20,222	241,597.2	0.81 (0.72, 0.91)	0.86 (0.76, 0.97)	285/19,085	229,024.4	0.83 (0.59, 1.18)	0.84 (0.59, 1.18)
1.00	803/10,649	126,634.1	0.95 (0.84, 1.07)	0.96 (0.85, 1.08)	1,229/50,367	609,763.7	0.91 (0.65, 1.27)	0.89 (0.64, 1.24)
Be physically active								
0.00	1,182/17,714	211,645.8	1.00 (reference)	1.00 (reference)	864/38,271	464,082.6	1.00 (reference)	1.00 (reference)
0.50	159/3,455	41,653.8	0.75 (0.63, 0.88)	0.79 (0.67, 0.94)	139/6,489	79,031.6	0.98 (0.82, 1.17)	0.99 (0.83, 1.18)
1.00	908/16,245	192,659.9	0.72 (0.66, 0.79)	0.77 (0.70, 0.84)	547/26,986	322,982.5	0.89 (0.80, 0.99)	0.89 (0.80, 0.99)
Maintain a healthy weight (body mass index)								
0.00	883/15,457	184,366.5	1.00 (reference)	1.00 (reference)	558/21,623	260,312.7	1.00 (reference)	1.00 (reference)
0.25	589/11,259	134,793.9	0.87 (0.78, 0.96)	0.89 (0.80, 0.98)	423/19,072	231,376.4	0.95 (0.83, 1.07)	0.96 (0.85, 1.09)
0.50	777/10,698	126,799.1	1.19 (1.08, 1.31)	1.21 (1.10, 1.33)	569/31,051	374,407.7	0.98 (0.87, 1.10)	1.01 (0.89, 1.14)
Maintain a healthy weight (waist circumference)								
0.00	686/10,805	129,739.0	1.00 (reference)	1.00 (reference)	440/14,795	179,679.4	1.00 (reference)	1.00 (reference)
0.50	1,563/26,609	316,220.6	1.01 (0.92, 1.10)	1.05 (0.96, 1.14)	1,110/56,951	686,417.3	0.96 (0.86, 1.07)	0.99 (0.89, 1.11)

HR, hazard ratio; CI, confidence interval.

1 Adjusted for education level (less than high school, high school, college or above and missing), Charlson comorbidity index (continuous), and total energy intake (tertiles).

## Supplementary materials

Supplementary Material 1. Operationalization of the Korean National Code Against Cancer in the HEXA study. N (%)

Supplementary Material 2. Hazard ratios (HRs) and 95% confidence intervals (CI) for cancer-specific mortality according to the Korean National Code Against Cancer adherence score categories.

Supplementary Material 3. Associations between adherence to individual components of the Korean National Code Against Cancer and cancer mortality.

Supplementary Material 4. Associations between adherence to individual components of the Korean National Code Against Cancer and lung cancer mortality.

Supplementary Material 5. Associations between adherence to individual components of the Korean National Code Against Cancer and stomach cancer mortality.

Supplementary Material 6. Associations between adherence to individual components of the Korean National Code Against Cancer and colorectal cancer mortality.

Supplementary Material 7. Associations between adherence to individual components of the Korean National Code Against Cancer and prostate / breast cancer mortality.

Supplementary Material 8. Hazard ratios (HRs) and 95% confidence intervals (CIs) for sensitivity analysis of mortality according to the Korean National Code Against Cancer adherence score categories.
