# Supplementary material for: Adherence to the Korean National Code Against Cancer and mortality: a prospective cohort study from the Health Examinees-Gem study
Source: Epidemiol Health. 2025 May 9;47:e2025026. doi: 10.4178/epih.e2025026 (PMC12425855; doi:10.4178/epih.e2025026)
Supplement: Supplementary Material 1. — Operationalization of the Korean National Code Against Cancer in the HEXA study. N (%) [file epih-47-e2025026-Supplementary-1.docx]

Supplementary Material 1. Operationalization of the Korean National Code Against Cancer in the HEXA study. N (%)

| **Korean National Code Against Cancer** | **Operationalization of Recommendations** | **Points** | **Men** | **Women** |
| --- | --- | --- | --- | --- |
| Limit smoking: Smoking status | Never | 1.0 | 10069 (26.9) | 69193 (96.4) |
|  | Former | 0.5 | 15464 (41.3) | 906 (1.3) |
|  | Current | 0.0 | 11881 (31.8) | 1647 (2.3) |
| Eat plenty of vegetables and fruits: Fruits and vegetables (g/day) | ≥400 | 1.0 | 6075 (16.2) | 10570 (14.7) |
|  | 200–<400 | 0.5 | 16727 (44.7) | 31056 (43.3) |
|  | <200 | 0.0 | 14612 (39.1) | 30120 (42.0) |
| Eat food without salty: Total Sodium intake (g/mg) | 2300-<4100 | 1.0 | 16070 (42.9) | 27225 (38.0) |
|  | 700–<2300, 4100–<5000 | 0.5 | 17923 (47.9) | 38247 (53.3) |
|  | <700, ≥5000 | 0.0 | 3421 (9.2) | 6274 (8.7) |
| Limit alcohol consumption: Total ethanol (g/day) | 0 | 1.0 | 10649 (28.5) | 50367 (70.2) |
|  | >0–≤28 (2 drinks) men and ≤14 (1 drink) women | 0.5 | 20222 (54.1) | 19085 (26.6) |
|  | >28 (2 drinks) men and >14 (1 drink) women | 0.0 | 6543 (17.5) | 2294 (3.2) |
| 5 or more times a week, 30 minutes or more a day, walking or exercising enough to sweat: Total moderate-vigorous physical activity (min/wk) | ≥150 | 1.0 | 16245 (43.4) | 26986 (37.6) |
|  | 75–<150 | 0.5 | 3455 (9.2) | 6489 (9.0) |
|  | <75 | 0.0 | 17714 (47.4) | 38271 (53.3) |
| Maintain a healthy weight: BMI(kg/m2, Waist circumference (cm)) | 18.5-22.9 | 0.50 | 10698 (28.6) | 31051 (43.3) |
|  | 23.0-24.9 | 0.25 | 11259 (30.1) | 19072 (26.6) |
|  | <18.5 or ≥25 | 0.00 | 15457 (41.3) | 21623 (30.1) |
|  | Men: <90, Women: <85 | 0.5 | 26609 (71.1) | 56951 (79.4) |
|  | Men: ≥90, Women: ≥85 | 0.0 | 10805 (28.9) | 14795 (20.6) |
| Hepatitis B and cervical cancer vaccination | Not included |  |  |  |
| Have a safe sexual health | Not included |  |  |  |
| Observe safety and health rules | Not included |  |  |  |
| Have a cancer screening | Not included |  |  |  |
