# Supplementary material for: Adherence to the Korean National Code Against Cancer and mortality: a prospective cohort study from the Health Examinees-Gem study
Source: Epidemiol Health. 2025 May 9;47:e2025026. doi: 10.4178/epih.e2025026 (PMC12425855; doi:10.4178/epih.e2025026)
Supplement: Supplementary Material 2. — Hazard ratios (HRs) and 95% confidence intervals (CI) for cancer-specific mortality according to the Korean National Code Against Cancer adherence score categories. [file epih-47-e2025026-Supplementary-2.docx]

Supplementary Material 2. Hazard ratios (HRs) and 95% confidence intervals (CI) for cancer-specific mortality according to the Korean National Code Against Cancer adherence score categories.

|  | Men (n=37414) | | | | |  | Women (n=71746) | | | | |
| --- | --- | --- | --- | --- | --- | --- | --- | --- | --- | --- | --- |
|  | Tertile 1 | Tertile 2 | Tertile 3 | p for trend | Continuous  (per 1point increase in score) |  | Tertile 1 | Tertile 2 | Tertile 3 | p for trend | Continuous  (per 1point increase in score) |
| Score range | 0.00-2.50 | 2.75-3.50 | 3.75-6.00 |  |  |  | 0.50-3.50 | 3.75-4.25 | 4.50-6.00 |  |  |
| Person year | 144022.5 | 166685.0 | 135252.1 |  |  |  | 346824.9 | 229565.5 | 289706.3 |  |  |
| Lung cancer |  |  |  |  |  |  |  |  |  |  |  |
| no.of deaths | 101 | 103 | 64 |  |  |  | 59 | 37 | 42 |  |  |
| Crude HR (95%CI) | 1.00 | 0.74  (0.57-0.98) | 0.48  (0.35-0.66) | <.001 | 0.74  (0.65-0.83) |  | 1.00 | 0.98  (0.65-1.48) | 0.90  (0.61-1.34) | 0.608 | 0.94  (0.78-1.12) |
| Multivariable adjusted HR (95%CI) | 1.00 | 0.86  (0.58-1.00) | 0.50  (0.37-0.69) | <.001 | 0.75  (0.66-0.85) |  | 1.00 | 0.94  (0.62-1.43) | 0.84  (0.56-1.25) | 0.392 | 0.90  (0.75-1.08) |
| Stomach cancer |  |  |  |  |  |  |  |  |  |  |  |
| no.of deaths | 19 | 26 | 24 |  |  |  | 19 | 12 | 22 |  |  |
| Crude HR (95%CI) | 1.00 | 1.05  (0.58-1.92) | 1.07  (0.58-1.97) | 0.837 | 0.98  (0.79-1.22) |  | 1.00 | 0.97  (0.47-2.01) | 1.43  (0.77-2.65) | 0.256 | 1.28  (0.93-1.75) |
| Multivariable adjusted HR (95%CI) | 1.00 | 1.12  (0.61-2.06) | 1.23  (0.67-2.25) | 0.519 | 1.04  (0.84-1.30) |  | 1.00 | 0.99  (0.48-2.02) | 1.46  (0.77-2.77) | 0.247 | 1.30  (0.94-1.81) |
| Colorectal cancer |  |  |  |  |  |  |  |  |  |  |  |
| no.of deaths | 19 | 29 | 27 |  |  |  | 38 | 23 | 20 |  |  |
| Crude HR (95%CI) | 1.00 | 1.18  (0.66-2.10) | 1.20  (0.67-2.17) | 0.541 | 1.01  (0.82-1.23) |  | 1.00 | 0.92  (0.55-1.56) | 0.64  (0.37-1.11) | 0.115 | 0.83  (0.66-1.03) |
| Multivariable adjusted HR (95%CI) | 1.00 | 1.14  (0.63-2.06) | 1.15  (0.63-2.11) | 0.644 | 0.99  (0.80-1.21) |  | 1.00 | 0.89  (0.53-1.50) | 0.60  (0.34-1.05) | 0.073 | 0.80  (0.63-1.01) |
| Prostate / Breast cancer |  |  |  |  |  |  |  |  |  |  |  |
| no.of deaths | 13 | 10 | 13 |  |  |  | 35 | 13 | 37 |  |  |
| Crude HR (95%CI) | 1.00 | 0.55  (0.24-1.26) | 0.73  (0.34-1.60) | 0.414 | 0.92  (0.65-1.32) |  | 1.00 | 0.57  (0.30-1.08) | 1.30  (0.82-2.07) | 0.287 | 1.02  (0.78-1.33) |
| Multivariable adjusted HR (95%CI) | 1.00 | 0.51  (0.22-1.19) | 0.64  (0.27-1.49) | 0.261 | 0.87  (0.60-1.27) |  | 1.00 | 0.56  (0.30-1.06) | 1.25  (0.77-2.03) | 0.374 | 0.99  (0.76-1.30) |

^a^ Adjusted for education level (less than high school, high school, college or above and missing), Charlson Comorbidity Index (continuous), and total energy intake (tertiles).
