# Supplementary material for: Adherence to the Korean National Code Against Cancer and mortality: a prospective cohort study from the Health Examinees-Gem study
Source: Epidemiol Health. 2025 May 9;47:e2025026. doi: 10.4178/epih.e2025026 (PMC12425855; doi:10.4178/epih.e2025026)
Supplement: Supplementary Material 3. — Associations between adherence to individual components of the Korean National Code Against Cancer and cancer mortality. [file epih-47-e2025026-Supplementary-3.docx]

Supplementary Material 3. Associations between adherence to individual components of the Korean National Code Against Cancer and cancer mortality.

|  |  |  | Men (n=37414) |  |  |  |  | Women (n=71746) |  |
| --- | --- | --- | --- | --- | --- | --- | --- | --- | --- |
| Components of Korean National Code Against Cancer score | No.of deaths /total participants | Person year | Crude HR  (95%CI) | Adjusted HR(95%CI) ^a^ |  | No.of deaths /total participants | Person year | Crude HR (95%CI) | Adjusted HR(95%CI) ^a^ |
| Smoking status |  |  |  |  |  |  |  |  |  |
| 0 | 397/11881 | 140682.0 | 1.00 | 1.00 |  | 28/1647 | 19434.1 | 1.00 | 1.00 |
| 0.5 | 445/15464 | 183111.9 | 0.59 (0.51-0.67) | 0.58 (0.51-0.67) |  | 20/906 | 10794.9 | 1.14 (0.64-2.01) | 1.13 (0.64-2.00) |
| 1 | 199/10069 | 122165.7 | 0.40 (0.34-0.48) | 0.42 (0.35-0.49) |  | 824/69193 | 835867.7 | 0.52 (0.36-0.76) | 0.52 (0.36-0.76) |
| Eat plenty of vegetables and fruits |  |  |  |  |  |  |  |  |  |
| 0 | 423/14612 | 173614.7 | 1.00 | 1.00 |  | 378/30120 | 361181.5 | 1.00 | 1.00 |
| 0.5 | 459/16727 | 198806.0 | 0.97 (0.85-1.11) | 0.97 (0.85-1.11) |  | 365/31056 | 373488.7 | 0.95 (0.82-1.09) | 0.93 (0.80-1.08) |
| 1 | 159/6075 | 73538.9 | 0.91 (0.76-1.10) | 0.92 (0.76-1.12) |  | 129/10570 | 131426.5 | 0.94 (0.77-1.15) | 0.91 (0.73-1.12) |
| Eat food without salty |  |  |  |  |  |  |  |  |  |
| 0 | 106/3421 | 41618.3 | 1.00 | 1.00 |  | 84/6274 | 78129.5 | 1.00 | 1.00 |
| 0.5 | 490/17923 | 212675.3 | 0.92 (0.74-1.13) | 0.91 (0.73-1.12) |  | 478/38247 | 458538.5 | 1.01 (0.80-1.28) | 1.02 (0.81-1.29) |
| 1 | 445/16070 | 191666.0 | 0.97 (0.78-1.19) | 0.96 (0.77-1.19) |  | 310/27225 | 329428.8 | 0.93 (0.73-1.19) | 0.93 (0.73-1.19) |
| Limit alcohol consumption |  |  |  |  |  |  |  |  |  |
| 0 | 173/6543 | 77728.2 | 1.00 | 1.00 |  | 18/2294 | 27308.6 | 1.00 | 1.00 |
| 0.5 | 497/20222 | 241597.2 | 0.80 (0.68-0.95) | 0.84 (0.71-1.00) |  | 181/19085 | 229024.4 | 1.07 (0.66-1.74) | 1.06 (0.66-1.73) |
| 1 | 371/10649 | 126634.1 | 0.93 (0.78-1.12) | 0.94 (0.78-1.13) |  | 673/50367 | 609763.7 | 1.05 (0.66-1.68) | 1.01 (0.63-1.62) |
| Be physically active |  |  |  |  |  |  |  |  |  |
| 0 | 536/17714 | 211645.8 | 1.00 | 1.00 |  | 458/38271 | 464082.6 | 1.00 | 1.00 |
| 0.5 | 73/3455 | 41653.8 | 0.76 (0.60-0.97) | 0.78 (0.61-1.00) |  | 82/6489 | 79031.6 | 1.07 (0.85-1.36) | 1.06 (0.84-1.34) |
| 1 | 432/16245 | 192659.9 | 0.75 (0.66-0.85) | 0.77 (0.68-0.88) |  | 332/26986 | 322982.5 | 1.01 (0.88-1.16) | 0.99 (0.86-1.14) |
| Be a healthy weight(BMI) |  |  |  |  |  |  |  |  |  |
| 0 | 408/15457 | 184366.5 | 1.00 | 1.00 |  | 296/21623 | 260312.7 | 1.00 | 1.00 |
| 0.25 | 287/11259 | 134793.9 | 0.91 (0.78-1.06) | 0.93 (0.80-1.08) |  | 258/19072 | 231376.4 | 1.07 (0.90-1.26) | 1.08 (0.91-1.27) |
| 0.5 | 346/10698 | 126799.1 | 1.14 (0.99-1.32) | 1.17 (1.01-1.35) |  | 318/31051 | 374407.7 | 0.99 (0.85-1.17) | 1.01 (0.85-1.18) |
| Be a healthy weight(Waist circumference)) |  |  |  |  |  |  |  |  |  |
| 0 | 330/10805 | 129739.0 | 1.00 | 1.00 |  | 229/14795 | 179679.4 | 1.00 | 1.00 |
| 0.5 | 711/26609 | 316220.6 | 0.95 (0.84-1.09) | 1.00 (0.88-1.14) |  | 643/56951 | 686417.3 | 1.02 (0.88-1.19) | 1.04 (0.89-1.22) |

^a^ Adjusted for education level (less than high school, high school, college or above and missing), Charlson Comorbidity Index (continuous), and total energy intake (tertiles).
