# Supplementary material for: Adherence to the Korean National Code Against Cancer and mortality: a prospective cohort study from the Health Examinees-Gem study
Source: Epidemiol Health. 2025 May 9;47:e2025026. doi: 10.4178/epih.e2025026 (PMC12425855; doi:10.4178/epih.e2025026)
Supplement: Supplementary Material 4. — Associations between adherence to individual components of the Korean National Code Against Cancer and lung cancer mortality. [file epih-47-e2025026-Supplementary-4.docx]

Supplementary Material 4. Associations between adherence to individual components of the Korean National Code Against Cancer and lung cancer mortality.

|  |  |  | Men (n=37414) |  |  |  |  | Women (n=71746) |  |
| --- | --- | --- | --- | --- | --- | --- | --- | --- | --- |
| Components of Korean National Code Against Cancer score | No.of deaths /total participants | Person year | Crude HR  (95%CI) | Adjusted HR(95%CI) ^a^ |  | No.of deaths /total participants | Person year | Crude HR (95%CI) | Adjusted HR(95%CI) ^a^ |
| Smoking status |  |  |  |  |  |  |  |  |  |
| 0 | 139/11881 | 140682.0 | 1.00 | 1.00 |  | 9/1647 | 19434.1 | 1.00 | 1.00 |
| 0.5 | 103/15464 | 183111.9 | 0.35 (0.27-0.45) | 0.35 (0.27-0.46) |  | 3/906 | 10794.9 | 0.51 (0.14-1.86) | 0.49 (0.13-1.80) |
| 1 | 26/10069 | 122165.7 | 0.13 (0.09-0.21) | 0.13 (0.09-0.21) |  | 126/69193 | 835867.7 | 0.23 (0.12-0.46) | 0.22 (0.11-0.44) |
| Eat plenty of vegetables and fruits |  |  |  |  |  |  |  |  |  |
| 0 | 103/14612 | 173614.7 | 1.00 | 1.00 |  | 60/30120 | 361181.5 | 1.00 | 1.00 |
| 0.5 | 127/16727 | 198806.0 | 1.11 (0.85-1.43) | 1.09 (0.84-1.42) |  | 64/31056 | 373488.7 | 1.05 (0.74-1.49) | 0.95 (0.66-1.37) |
| 1 | 38/6075 | 73538.9 | 0.91 (0.63-1.32) | 0.88 (0.59-1.32) |  | 14/10570 | 131426.5 | 0.65 (0.36-1.16) | 0.53 (0.29-0.98) |
| Eat food without salty |  |  |  |  |  |  |  |  |  |
| 0 | 23/3421 | 41618.3 | 1.00 | 1.00 |  | 15/6274 | 78129.5 | 1.00 | 1.00 |
| 0.5 | 126/17923 | 212675.3 | 1.09 (0.70-1.70) | 1.11 (0.71-1.74) |  | 74/38247 | 458538.5 | 0.88 (0.51-1.54) | 0.90 (0.51-1.57) |
| 1 | 119/16070 | 191666.0 | 1.20 (0.77-1.89) | 1.21 (0.77-1.90) |  | 49/27225 | 329428.8 | 0.84 (0.47-1.49) | 0.79 (0.44-1.41) |
| Limit alcohol consumption |  |  |  |  |  |  |  |  |  |
| 0 | 40/6543 | 77728.2 | 1.00 | 1.00 |  | 3/2294 | 27308.6 | 1.00 | 1.00 |
| 0.5 | 135/20222 | 241597.2 | 0.90 (0.63-1.28) | 0.96 (0.67-1.37) |  | 25/19085 | 229024.4 | 0.84 (0.25-2.78) | 0.82 (0.25-2.71) |
| 1 | 93/10649 | 126634.1 | 0.92 (0.64-1.32) | 0.95 (0.66-1.38) |  | 110/50367 | 609763.7 | 0.88 (0.28-2.81) | 0.86 (0.27-2.74) |
| Be physically active |  |  |  |  |  |  |  |  |  |
| 0 | 144/17714 | 211645.8 | 1.00 | 1.00 |  | 74/38271 | 464082.6 | 1.00 | 1.00 |
| 0.5 | 19/3455 | 41653.8 | 0.76 (0.47-1.23) | 0.81 (0.50-1.31) |  | 8/6489 | 79031.6 | 0.66 (0.32-1.36) | 0.65 (0.32-1.35) |
| 1 | 105/16245 | 192659.9 | 0.66 (0.51-0.84) | 0.69 (0.54-0.90) |  | 56/26986 | 322982.5 | 1.06 (0.74-1.49) | 1.05 (0.74-1.49) |
| Be a healthy weight(BMI) |  |  |  |  |  |  |  |  |  |
| 0 | 92/15457 | 184366.5 | 1.00 | 1.00 |  | 37/21623 | 260312.7 | 1.00 | 1.00 |
| 0.25 | 80/11259 | 134793.9 | 1.11 (0.82-1.49) | 1.13 (0.84-1.52) |  | 43/19072 | 231376.4 | 1.46 (0.94-2.26) | 1.47 (0.95-2.27) |
| 0.5 | 96/10698 | 126799.1 | 1.38 (1.03-1.31) | 1.40 (1.05-1.87) |  | 58/31051 | 374407.7 | 1.58 (1.04-2.41) | 1.60 (1.05-2.44) |
| Be a healthy weight(Waist circumference)) |  |  |  |  |  |  |  |  |  |
| 0 | 74/10805 | 129739.0 | 1.00 | 1.00 |  | 38/14795 | 179679.4 | 1.00 | 1.00 |
| 0.5 | 194/26609 | 316220.6 | 1.19 (0.91-1.55) | 1.23 (0.94-1.61) |  | 100/56951 | 686417.3 | 1.04 (0.71-1.52) | 1.05 (0.71-1.55) |

^a^ Adjusted for education level (less than high school, high school, college or above and missing), Charlson Comorbidity Index (continuous), and total energy intake (tertiles).
