# Supplementary material for: Adherence to the Korean National Code Against Cancer and mortality: a prospective cohort study from the Health Examinees-Gem study
Source: Epidemiol Health. 2025 May 9;47:e2025026. doi: 10.4178/epih.e2025026 (PMC12425855; doi:10.4178/epih.e2025026)
Supplement: Supplementary Material 5. — Associations between adherence to individual components of the Korean National Code Against Cancer and stomach cancer mortality. [file epih-47-e2025026-Supplementary-5.docx]

Supplementary Material 5. Associations between adherence to individual components of the Korean National Code Against Cancer and stomach cancer mortality.

|  |  |  | Men (n=37414) |  |  |  |  | Women (n=71746) |  |
| --- | --- | --- | --- | --- | --- | --- | --- | --- | --- |
| Components of Korean National Code Against Cancer score | No.of deaths /total participants | Person year | Crude HR  (95%CI) | Adjusted HR(95%CI) ^a^ |  | No.of deaths /total participants | Person year | Crude HR (95%CI) | Adjusted HR(95%CI) ^a^ |
| Smoking status |  |  |  |  |  |  |  |  |  |
| 0 | 19/11881 | 140682.0 | 1.00 | 1.00 |  | 0/1647 | 19434.1 | 1.00 | 1.00 |
| 0.5 | 31/15464 | 183111.9 | 0.92 (0.50-1.67) | 0.92 (0.51-1.66) |  | 0/906 | 10794.9 | 1.00 (0.90-1.12) | 1.00 (0.88-1.13) |
| 1 | 19/10069 | 122165.7 | 0.86 (0.45-1.64) | 0.91 (0.48-1.72) |  | 53/69193 | 835867.7 | - | - |
| Eat plenty of vegetables and fruits |  |  |  |  |  |  |  |  |  |
| 0 | 27/14612 | 173614.7 | 1.00 | 1.00 |  | 20/30120 | 361181.5 | 1.00 | 1.00 |
| 0.5 | 34/16727 | 198806.0 | 1.12 (0.68-1.86) | 1.24 (0.76-2.02) |  | 29/31056 | 373488.7 | 1.42 (0.80-2.50) | 1.49 (0.84-2.64) |
| 1 | 8/6075 | 73538.9 | 0.72 (0.33-1.58) | 0.88 (0.38-2.00) |  | 4/10570 | 131426.5 | 0.55 (0.19-1.62) | 0.56 (0.18-1.69) |
| Eat food without salty |  |  |  |  |  |  |  |  |  |
| 0 | 7/3421 | 41618.3 | 1.00 | 1.00 |  | 3/6274 | 78129.5 | 1.00 | 1.00 |
| 0.5 | 32/17923 | 212675.3 | 0.91 (0.40-2.06) | 0.87 (0.37-2.01) |  | 26/38247 | 458538.5 | 1.51 (0.46-4.96) | 1.61 (0.49-5.26) |
| 1 | 30/16070 | 191666.0 | 0.98 (0.43-2.23) | 1.01 (0.44-2.32) |  | 24/27225 | 329428.8 | 1.96 (0.59-6.48) | 2.25 (0.67-7.60) |
| Limit alcohol consumption |  |  |  |  |  |  |  |  |  |
| 0 | 8/6543 | 77728.2 | 1.00 | 1.00 |  | 0/2294 | 27308.6 | 1.00 | 1.00 |
| 0.5 | 37/20222 | 241597.2 | 1.31 (0.61-2.82) | 1.39 (0.64-3.01) |  | 9/19085 | 229024.4 | - | - |
| 1 | 24/10649 | 126634.1 | 1.37 (0.60-3.09) | 1.35 (0.59-3.06) |  | 44/50367 | 609763.7 | - | - |
| Be physically active |  |  |  |  |  |  |  |  |  |
| 0 | 32/17714 | 211645.8 | 1.00 | 1.00 |  | 29/38271 | 464082.6 | 1.00 | 1.00 |
| 0.5 | 5/3455 | 41653.8 | 0.86 (0.33-2.19) | 0.91 (0.35-2.33) |  | 5/6489 | 79031.6 | 1.03 (0.40-2.65) | 1.00 (0.36-2.56) |
| 1 | 32/16245 | 192659.9 | 0.96 (0.58-1.57) | 1.02 (0.61-1.70) |  | 19/26986 | 322982.5 | 0.94 (0.53-1.66) | 0.89 (0.50-1.59) |
| Be a healthy weight(BMI) |  |  |  |  |  |  |  |  |  |
| 0 | 26/15457 | 184366.5 | 1.00 | 1.00 |  | 14/21623 | 260312.7 | 1.00 | 1.00 |
| 0.25 | 17/11259 | 134793.9 | 0.85 (0.46-1.56) | 0.87 (0.47-1.60) |  | 15/19072 | 231376.4 | 1.27 (0.62-2.63) | 1.28 (0.62-2.67) |
| 0.5 | 26/10698 | 126799.1 | 1.36 (0.79-2.34) | 1.37 (0.79-2.37) |  | 24/31051 | 374407.7 | 1.40 (0.71-2.79) | 1.42 (0.70-2.85) |
| Be a healthy weight(Waist circumference)) |  |  |  |  |  |  |  |  |  |
| 0 | 24/10805 | 129739.0 | 1.00 | 1.00 |  | 8/14795 | 179679.4 | 1.00 | 1.00 |
| 0.5 | 45/26609 | 316220.6 | 0.82 (0.50-1.34) | 0.85 (0.51-1.42) |  | 45/56951 | 686417.3 | 1.81 (0.82-4.04) | 1.85 (0.81-4.21) |

^a^ Adjusted for education level (less than high school, high school, college or above and missing), Charlson Comorbidity Index (continuous), and total energy intake (tertiles).
