# Supplementary material for: Adherence to the Korean National Code Against Cancer and mortality: a prospective cohort study from the Health Examinees-Gem study
Source: Epidemiol Health. 2025 May 9;47:e2025026. doi: 10.4178/epih.e2025026 (PMC12425855; doi:10.4178/epih.e2025026)
Supplement: Supplementary Material 6. — Associations between adherence to individual components of the Korean National Code Against Cancer and colorectal cancer mortality. [file epih-47-e2025026-Supplementary-6.docx]

Supplementary Material 6. Associations between adherence to individual components of the Korean National Code Against Cancer and colorectal cancer mortality.

|  |  |  | Men (n=37414) |  |  |  |  | Women (n=71746) |  |
| --- | --- | --- | --- | --- | --- | --- | --- | --- | --- |
| Components of Korean National Code Against Cancer score | No.of deaths /total participants | Person year | Crude HR  (95%CI) | Adjusted HR(95%CI) ^a^ |  | No.of deaths /total participants | Person year | Crude HR (95%CI) | Adjusted HR(95%CI) ^a^ |
| Smoking status |  |  |  |  |  |  |  |  |  |
| 0 | 21/11881 | 140682.0 | 1.00 | 1.00 |  | 4/1647 | 19434.1 | 1.00 | 1.00 |
| 0.5 | 36/15464 | 183111.9 | 0.94 (0.54-1.63) | 0.91 (0.52-1.58) |  | 2/906 | 10794.9 | 0.83 (0.15-4.47) | 0.81 (0.15-4.40) |
| 1 | 18/10069 | 122165.7 | 0.73 (0.38-1.40) | 0.75 (0.39-1.44) |  | 75/69193 | 835867.7 | 0.36 (0.13-0.98) | 0.35 (0.13-0.95) |
| Eat plenty of vegetables and fruits |  |  |  |  |  |  |  |  |  |
| 0 | 27/14612 | 173614.7 | 1.00 | 1.00 |  | 37/30120 | 361181.5 | 1.00 | 1.00 |
| 0.5 | 36/16727 | 198806.0 | 1.19 (0.72-1.96) | 1.12 (0.68-1.84) |  | 32/31056 | 373488.7 | 0.84 (0.53-1.35) | 0.78 (0.48-1.29) |
| 1 | 12/6075 | 73538.9 | 1.09 (0.55-2.14) | 0.97 (0.48-1.98) |  | 12/10570 | 131426.5 | 0.89 (0.46-1.70) | 0.80 (0.39-1.63) |
| Eat food without salty |  |  |  |  |  |  |  |  |  |
| 0 | 7/3421 | 41618.3 | 1.00 | 1.00 |  | 7/6274 | 78129.5 | 1.00 | 1.00 |
| 0.5 | 32/17923 | 212675.3 | 0.90 (0.40-2.03) | 0.89 (0.84-1.12) |  | 43/38247 | 458538.5 | 1.08 (0.49-2.40) | 1.05 (0.46-2.39) |
| 1 | 36/16070 | 191666.0 | 1.17 (0.52-2.63) | 1.12 (0.49-2.54) |  | 31/27225 | 329428.8 | 1.10 (0.49-2.50) | 1.02 (0.44-2.40) |
| Limit alcohol consumption |  |  |  |  |  |  |  |  |  |
| 0 | 12/6543 | 77728.2 | 1.00 | 1.00 |  | 2/2294 | 27308.6 | 1.00 | 1.00 |
| 0.5 | 36/20222 | 241597.2 | 0.86 (0.45-1.64) | 0.88 (0.45-1.70) |  | 19/19085 | 229024.4 | 1.07 (0.25-4.60) | 1.05 (0.25-4.49) |
| 1 | 27/10649 | 126634.1 | 1.02 (0.52-2.02) | 1.01 (0.51-2.01) |  | 60/50367 | 609763.7 | 1.00 (0.24-4.10) | 0.98 (0.24-4.00) |
| Be physically active |  |  |  |  |  |  |  |  |  |
| 0 | 34/17714 | 211645.8 | 1.00 | 1.00 |  | 46/38271 | 464082.6 | 1.00 | 1.00 |
| 0.5 | 5/3455 | 41653.8 | 0.81 (0.32-2.06) | 0.78 (0.30-2.04) |  | 11/6489 | 79031.6 | 1.42 (0.73-2.75) | 1.41 (0.73-2.73) |
| 1 | 36/16245 | 192659.9 | 1.00 (0.63-1.61) | 0.96 (0.58-1.57) |  | 24/26986 | 322982.5 | 0.73 (0.44-1.20) | 0.73 (0.44-1.19) |
| Be a healthy weight(BMI) |  |  |  |  |  |  |  |  |  |
| 0 | 29/15457 | 184366.5 | 1.00 | 1.00 |  | 31/21623 | 260312.7 | 1.00 | 1.00 |
| 0.25 | 19/11259 | 134793.9 | 0.85 (0.48-1.52) | 0.89 (0.50-1.59) |  | 20/19072 | 231376.4 | 0.77 (0.44-1.36) | 0.77 (0.44-1.35) |
| 0.5 | 27/10698 | 126799.1 | 1.28 (0.75-2.17) | 1.34 (0.79-2.28) |  | 30/31051 | 374407.7 | 0.81 (0.49-1.34) | 0.81 (0.49-1.34) |
| Be a healthy weight(Waist circumference)) |  |  |  |  |  |  |  |  |  |
| 0 | 28/10805 | 129739.0 | 1.00 | 1.00 |  | 21/14795 | 179679.4 | 1.00 | 1.00 |
| 0.5 | 47/26609 | 316220.6 | 0.74 (0.46-1.18) | 0.80 (0.50-1.26) |  | 60/56951 | 686417.3 | 0.94 (0.56-1.56) | 0.94 (0.57-1.56) |

^a^ Adjusted for education level (less than high school, high school, college or above and missing), Charlson Comorbidity Index (continuous), and total energy intake (tertiles).
