# Supplementary material for: Adherence to the Korean National Code Against Cancer and mortality: a prospective cohort study from the Health Examinees-Gem study
Source: Epidemiol Health. 2025 May 9;47:e2025026. doi: 10.4178/epih.e2025026 (PMC12425855; doi:10.4178/epih.e2025026)
Supplement: Supplementary Material 7. — Associations between adherence to individual components of the Korean National Code Against Cancer and prostate / breast cancer mortality. [file epih-47-e2025026-Supplementary-7.docx]

Supplementary Material 7. Associations between adherence to individual components of the Korean National Code Against Cancer and prostate / breast cancer mortality.

|  |  |  | Men (n=37414) |  |  |  |  | Women (n=71746) |  |
| --- | --- | --- | --- | --- | --- | --- | --- | --- | --- |
| Components of Korean National Code Against Cancer score | No.of deaths /total participants | Person year | Crude HR  (95%CI) | Adjusted HR(95%CI) ^a^ |  | No.of deaths /total participants | Person year | Crude HR (95%CI) | Adjusted HR(95%CI) ^a^ |
| Smoking status |  |  |  |  |  |  |  |  |  |
| 0 | 10/11881 | 140682.0 | 1.00 | 1.00 |  | 1/1647 | 19434.1 | 1.00 | 1.00 |
| 0.5 | 15/15464 | 183111.9 | 0.69 (0.32-1.53) | 0.67 (0.30-1.48) |  | 3/906 | 10794.9 | 5.09 (0.53-48.78) | 4.83 (0.51-46.25) |
| 1 | 11/10069 | 122165.7 | 0.76 (0.32-1.80) | 0.76 (0.32-1.83) |  | 81/69193 | 835867.7 | 1.69 (0.24-12.15) | 1.70 (0.24-12.16) |
| Eat plenty of vegetables and fruits |  |  |  |  |  |  |  |  |  |
| 0 | 12/14612 | 173614.7 | 1.00 | 1.00 |  | 34/30120 | 361181.5 | 1.00 | 1.00 |
| 0.5 | 16/16727 | 198806.0 | 1.20 (0.57-2.54) | 1.07 (0.50-2.28) |  | 30/31056 | 373488.7 | 0.86 (0.53-1.41) | 0.91 (0.55-1.51) |
| 1 | 8/6075 | 73538.9 | 1.65 (0.68-4.03) | 1.25 (0.52-3.02) |  | 21/10570 | 131426.5 | 1.71 (0.99-2.94) | 1.85 (1.06-3.23) |
| Eat food without salty |  |  |  |  |  |  |  |  |  |
| 0 | 6/3421 | 41618.3 | 1.00 | 1.00 |  | 9/6274 | 78129.5 | 1.00 | 1.00 |
| 0.5 | 12/17923 | 212675.3 | 0.40 (0.15-1.08) | 0.45 (0.17-1.23) |  | 45/38247 | 458538.5 | 0.87 (0.42-1.77) | 0.88 (0.43-1.79) |
| 1 | 18/16070 | 191666.0 | 0.71 (0.28-1.78) | 0.74 (0.29-1.85) |  | 31/27225 | 329428.8 | 0.84 (0.40-1.76) | 0.88 (0.41-1.88) |
| Limit alcohol consumption |  |  |  |  |  |  |  |  |  |
| 0 | 6/6543 | 77728.2 | 1.00 | 1.00 |  | 2/2294 | 27308.6 | 1.00 | 1.00 |
| 0.5 | 17/20222 | 241597.2 | 0.73 (0.29-1.84) | 0.74 (0.29-1.90) |  | 16/19085 | 229024.4 | 0.91 (0.21-3.96) | 0.92 (0.21-4.00) |
| 1 | 13/10649 | 126634.1 | 0.81 (0.31-2.13) | 0.81 (0.30-2.21) |  | 67/50367 | 609763.7 | 1.26 (0.31-5.10) | 1.16 (0.28-4.70) |
| Be physically active |  |  |  |  |  |  |  |  |  |
| 0 | 19/17714 | 211645.8 | 1.00 | 1.00 |  | 42/38271 | 464082.6 | 1.00 | 1.00 |
| 0.5 | 3/3455 | 41653.8 | 0.93 (0.27-3.19) | 0.87 (0.25-2.98) |  | 7/6489 | 79031.6 | 0.99 (0.45-2.21) | 0.93 (0.42-2.08) |
| 1 | 14/16245 | 192659.9 | 0.65 (0.33-1.30) | 0.58 (0.27-1.21) |  | 36/26986 | 322982.5 | 1.23 (0.78-1.93) | 1.12 (0.71-1.76) |
| Be a healthy weight(BMI) |  |  |  |  |  |  |  |  |  |
| 0 | 14/15457 | 184366.5 | 1.00 | 1.00 |  | 32/21623 | 260312.7 | 1.00 | 1.00 |
| 0.25 | 12/11259 | 134793.9 | 1.08 (0.50-2.34) | 1.14 (0.53-2.46) |  | 26/19072 | 231376.4 | 0.95 (0.57-1.60) | 0.97 (0.58-1.61) |
| 0.5 | 10/10698 | 126799.1 | 0.92 (0.41-2.06) | 0.99 (0.44-2.25) |  | 27/31051 | 374407.7 | 0.66 (0.39-1.10) | 0.67 (0.39-1.12) |
| Be a healthy weight(Waist circumference)) |  |  |  |  |  |  |  |  |  |
| 0 | 11/10805 | 129739.0 | 1.00 | 1.00 |  | 31/14795 | 179679.4 | 1.00 | 1.00 |
| 0.5 | 25/26609 | 316220.6 | 1.03 (0.51-2.10) | 1.14 (0.56-2.35) |  | 54/56951 | 686417.3 | 0.52 (0.33-0.82) | 0.52 (0.33-0.83) |

^a^ Adjusted for education level (less than high school, high school, college or above and missing), Charlson Comorbidity Index (continuous), and total energy intake (tertiles).
