# Supplementary material for: Adherence to the Korean National Code Against Cancer and mortality: a prospective cohort study from the Health Examinees-Gem study
Source: Epidemiol Health. 2025 May 9;47:e2025026. doi: 10.4178/epih.e2025026 (PMC12425855; doi:10.4178/epih.e2025026)
Supplement: Supplementary Material 8. — Hazard ratios (HRs) and 95% confidence intervals (CIs) for sensitivity analysis of mortality according to the Korean National Code Against Cancer adherence score categories. [file epih-47-e2025026-Supplementary-8.docx]

Supplementary Material 8. Hazard ratios (HRs) and 95% confidence intervals (CIs) for sensitivity analysis of mortality according to the Korean National Code Against Cancer adherence score categories.

|  |  |  |  | All-cause mortality |  |  | Cancer mortality |  |  | CVD mortality |  |
| --- | --- | --- | --- | --- | --- | --- | --- | --- | --- | --- | --- |
| Adherence score  (range) | Person year | No.of total subjects | No.of cases | Crude  HR(95%CI) | Adjusted HR(95%CI) ^a^ | No.of cases | Crude  HR(95%CI) | Adjusted HR(95%CI) ^a^ | No.of cases | Crude  HR(95%CI) | Adjusted HR(95%CI) ^a^ |
| **Men(n=37224)** |  |  |  |  |  |  |  |  |  |  |  |
| Tertile1 (0.00-2.50) | 143939.7 | 12047 | 749 | 1.00 | 1.00 | 353 | 1.00 | 1.00 | 131 | 1.00 | 1.00 |
| Tertile2 (2.75-3.50) | 166612.3 | 13902 | 736 | 0.75 (0.67-0.83) | 0.77 (0.70-0.86) | 339 | 0.73 (0.63-0.85) | 0.74 (0.64-0.86) | 117 | 0.67 (0.52-0.86) | 0.69 (0.54-0.89) |
| Tertile3 (3.75-6.00) | 135187.7 | 11275 | 574 | 0.63 (0.56-0.70) | 0.67 (0.60-0.75) | 269 | 0.62 (0.53-0.73) | 0.64 (0.55-0.75) | 88 | 0.54 (0.41-0.70) | 0.57 (0.43-0.75) |
| P trend |  |  |  | <.001 | <.001 |  | <.001 | <.001 |  | <.001 | <.001 |
| Continuous^b^ |  |  |  | 0.82 (0.78-0.86) | 0.85 (0.81-0.89) |  | 0.82 (0.76-0.87) | 0.83 (0.78-0.89) |  | 0.79 (0.71-0.88) | 0.81 (0.73-0.90) |
| **Women(n=71638)** |  |  |  |  |  |  |  |  |  |  |  |
| Tertile1 (0.50-3.50) | 346768.5 | 28718 | 654 | 1.00 | 1.00 | 354 | 1.00 | 1.00 | 108 | 1.00 | 1.00 |
| Tertile2 (3.75-4.25) | 229534.2 | 18957 | 356 | 0.85 (0.75-0.97) | 0.86 (0.76-0.98) | 210 | 0.92 (0.77-1.09) | 0.91 (0.76-1.08) | 52 | 0.78 (0.56-1.08) | 0.82 (0.59-1.14) |
| Tertile3 (4.50-6.00) | 289666.5 | 23963 | 432 | 0.83 (0.74-0.94) | 0.85 (0.75-0.96) | 261 | 0.91 (0.78-1.07) | 0.89 (0.76-1.05) | 50 | 0.60 (0.43-0.84) | 0.68 (0.48-0.95) |
| P trend |  |  |  | 0.002 | 0.008 |  | 0.248 | 0.166 |  | 0.003 | 0.023 |
| Continuous^b^ |  |  |  | 0.89 (0.84-0.95) | 0.90 (0.85-0.96) |  | 0.95 (0.88-1.03) | 0.94 (0.87-1.02) |  | 0.76 (0.65-0.88) | 0.81 (0.69-0.94) |

^a^ Adjusted for education level (less than high school, high school, college or above and missing), Charlson Comorbidity Index (continuous), and total energy intake (tertiles).
